# TGF-*β* and Hypoxia/Reoxygenation Promote Radioresistance of A549 Lung Cancer Cells through Activation of Nrf2 and EGFR

**DOI:** 10.1155/2016/6823471

**Published:** 2016-01-20

**Authors:** Sae-lo-oom Lee, Hwani Ryu, A-rang Son, Bitna Seo, Jooyoung Kim, Seung-Youn Jung, Jie-Young Song, Sang-Gu Hwang, Jiyeon Ahn

**Affiliations:** Division of Radiation Cancer Research, Korea Institute of Radiological & Medical Sciences (KIRAMS), 75 Nowon-ro, Nowon-gu, Seoul 01812, Republic of Korea

## Abstract

Although many studies have examined the roles of hypoxia and transforming growth factor- (TGF-) *β* separately in the tumor microenvironment, the effects of simultaneous treatment with hypoxia/reoxygenation and TGF-*β* on tumor malignancy are unclear. Here, we investigated the effects of redox signaling and oncogenes on cell proliferation and radioresistance in A549 human lung cancer cells in the presence of TGF-*β* under hypoxia/reoxygenation conditions. Combined treatment with TGF-*β* and hypoxia activated epidermal growth factor receptor (EGFR) and nuclear factor (erythroid-derived 2)-like 2 (Nrf2), a redox-sensitive transcription factor. Interestingly, Nrf2 knockdown suppressed the effects of combined treatment on EGFR phosphorylation. In addition, blockade of EGFR signaling also suppressed induction of Nrf2 following combined treatment with hypoxia and TGF-*β*, indicating that the combined treatment induced positive crosstalk between Nrf2 and EGFR. TGF-*β* and hypoxia/reoxygenation increased the accumulation of reactive oxygen species (ROS), while treatment with N-acetyl-l-cysteine abolished the activation of Nrf2 and EGFR. Treatment with TGF-*β* under hypoxic conditions increased the proliferation of A549 cells compared with that after vehicle treatment. Moreover, cells treated with the combined treatment exhibited resistance to ionizing radiation (IR), and knockdown of Nrf2 increased IR-induced cell death under these conditions. Thus, taken together, our findings suggested that TGF-*β* and hypoxia/reoxygenation promoted tumor progression and radioresistance of A549 cells through ROS-mediated activation of Nrf2 and EGFR.

## 1. Introduction

Lung cancer is the leading cause of cancer-related death in both men and women in many countries. Non-small cell lung cancer (NSCLC) accounts for more than 80–85% of all lung cancer cases, and the predicted 5-year survival rate of patients with NSCLC is 15.9% in the United States [1–3]. Although many studies have examined the genetic and molecular features of lung cancer in order to develop improved treatments, including specific target therapy, the treatment of lung cancer still remains a challenging and complicated issue due to interactions between lung tumors and the surrounding microenvironment, which has a degree of vascularization and oxygenation. Radiotherapy (RT) is commonly used for the effective treatment of solid tumors, inducing DNA damage and cytotoxicity by generating reactive oxygen species (ROS). However, the application of RT is limited by the acquisition of radioresistance in cells, conferred by the tumor microenvironment.

The tumor microenvironment plays an important role in tumor malignancy and treatment resistance. Hypoxia, characterized by a state of low oxygenation within a solid tumor, is a common phenomenon influencing tumor growth and malignancy and can be affected by the features of the tumor microenvironment [4]. Additionally, transforming growth factor- (TGF-) *β*, another key factor in the tumor microenvironment, has been shown to be a mediator of the epithelial-to-mesenchymal transition (EMT), tumor metastasis, and immune escape, thereby affecting tumor progression. While many studies have characterized the effects of hypoxia and TGF-*β* separately in the tumor microenvironment, the effects of concurrent treatment with hypoxia and TGF-*β* on tumor malignancy are unclear.

Nuclear factor (erythroid-derived 2)-like 2 (Nrf2) is a redox-sensitive transcription factor that plays a role in the antioxidant-response element- (ARE-) driven cellular defense system and was originally identified as a tumor suppressor, as shown in Nrf2-knockout mice [5, 6]. Nrf2 and its target genes (e.g., heme oxygenase-1 (HO-1)], NAD(P)H:quinone oxidoreductase 1 (NQO-1), and sulfiredoxin-1 (Srx1)) protect not only normal cells but also cancer cells from oxidative stress. Under unstressed conditions in normal cells, Nrf2 is sequestered in the cytoplasm by its inhibitory protein Kelch-like ECH-associated protein 1 (Keap1), which functions as a substrate adapter protein for a Cul3-Rbx E3 ubiquitin ligase core complex, and subsequently undergoes proteasomal degradation [7]. In cancer tissues and cell lines, including lung, breast, and ovarian cancer cells, Nrf2 is highly expressed and activated due to either the oxidative microenvironment or genetic mutations resulting in loss of Keap1 function or gain of Nrf2 function as compared with that in normal cells [8–10]; thus, Nrf2 readily eliminates ROS from microenvironmental oxidative stressors, providing survival benefits. Intriguingly, recent emerging data have reported the oncogenic roles of Nrf2 in cancer. Indeed, several studies have shown that Nrf2 is induced under hypoxic conditions, and positive correlations between hypoxia-induced factor- (HIF-) 1*α* and Nrf2 have been observed in glioblastoma, head and neck cancer, NSCLC, and colon cancer cells [11–13]. However, the interactions of Nrf2 signaling in the microenvironment with hypoxia/reoxygenation and TGF-*β* signaling remain unclear.

Epidermal growth factor receptor (EGFR) is a well-known oncogenic tyrosine kinase that is highly activated in NSCLC cells. Upon ligand binding, EGFR is activated through homo- or heterodimerization with other ErbB family members, resulting in autophosphorylation and activation of downstream pathways. Activated EGFR subsequently undergoes internalization from the cell surface by endocytosis and is then either cycled back to the plasma membrane or targeted for c-Cbl-mediated degradation; both of those mechanisms can function to downregulate EGFR signaling [14]. EGFR is overexpressed in many cancers, and its activation is related to the tumor microenvironment; for example, oxidative stress-mediated activation of EGFR leads to failure of EGFR to undergo entry into early endosomes for subsequent degradation, resulting in prolonged activation of EGFR signaling, tumorigenesis, and malignancy [15, 16].

In this study, we aimed to elucidate the mechanisms mediating the effects of combined hypoxia/reoxygenation and TGF-*β* signaling on cancer-related phenotypes (e.g., proliferation and radioresistant potential) as a representative model mimicking the* in vivo* tumor microenvironment. Our findings demonstrated that hypoxia/reoxygenation and TGF-*β* were crucial factors in Nrf2-mediated tumor resistance, which was correlated with EGFR activation.

## 2. Materials and Methods

### 2.1. Cell Culture, Treatment with TGF-*β*, and Induction of Hypoxia

The human lung adenocarcinoma cell line A549 (American Type Culture Collection, Manassas, VA, USA) was maintained in RPMI 1640 supplemented with 10% fetal bovine serum (FBS; Gibco BRL, Grand Island, NY, USA) and 0.5% penicillin/streptomycin solution (Mediatech, Inc., Herndon, VA, USA) and was incubated at 37°C in a humidified atmosphere with 5% CO_2_. For TGF-*β* and hypoxia/reoxygenation treatment, culture medium was replaced with deoxygenated RPMI 1640 (Gibco-BRL) before induction of hypoxia, and 1 ng/mL of human recombinant TGF-*β*_1_ (R&D Systems, Minneapolis, MN, USA) was added to the medium; cells were then incubated at 37°C in a hypoxic chamber (Forma Scientific, Marietta, OH, USA). Deoxygenated medium was prepared prior to each experiment by equilibrating the medium with a hypoxic gas mixture containing 5% CO_2_ and 7% H_2_ and balanced with N_2_ at 37°C. The oxygen concentration in the hypoxic chamber was maintained at less than 1% and was monitored using an oxygen indicator (Forma Scientific).

### 2.2. Reagents and Antibodies

AG1478 and LY294002 were purchased from Sigma-Aldrich (St. Louis, MO, USA). Antibodies specific for Nrf2, Srx, EGFR, extracellular signal-regulated kinase (ERK), phospho-ERK, NADPH oxidase (NOX) 4, c-Jun N-terminal kinase (JNK; all from Santa Cruz Biotechnology, Santa Cruz, CA, USA), HO-1, NQO-1, phospho-EGFR, Akt, phospho-Akt, p38, phospho-p38, phospho-JNK (all from Cell Signaling Technology, Danvers, MA, USA), NOX1, NOX2, lamin B1 (all from Abcam, Cambridge, MA, USA), HIF-1*α* (BD), and GAPDH (AbFrontier, Seoul, Korea) were used in this study.

### 2.3. Preparation of Nuclear Extracts

Nuclear extracts were prepared as described previously [17]. Briefly, cells were washed three times with phosphate-buffered saline (PBS), harvested, resuspended in a hypotonic buffer (10 mM HEPES (pH 7.9), 1.5 mM MgCl_2_, 10 mM KCl, and 0.1% NP40 containing 1 *μ*g/mL leupeptin, 1 mM phenylmethylsulfonyl fluoride (PMSF), 20 mM NaF, 1 mM sodium pyrophosphate, and 1 mM Na_3_VO_4_) and incubated for 15 min on ice. Nuclei were pelleted by centrifugation at 3000 ×g for 15 min at 4°C, resuspended in two volumes of cold hypertonic buffer (10 mM HEPES (pH 8.0), 25% glycerol, 0.4 M NaCl, and 0.1 mM EDTA containing 1 *μ*g/mL leupeptin, 1 mM PMSF, 20 mM NaF, 1 mM sodium pyrophosphate, and 1 mM Na_3_VO_4_), and incubated for 30 min on ice. Nuclear debris was removed by centrifugation, and the supernatant was recovered as the nuclear extract. Protein concentrations were determined using a Bio-Rad Protein Assay (Bio-Rad, Hercules, CA, USA) following the manufacturer's standard protocol.

### 2.4. Western Blot

Cell lysates were prepared by extracting proteins with RIPA buffer (50 mM Tris-Cl (pH 7.4), 1% NP-40, 150 mM NaCl, and 1 mM EDTA) supplemented with protease inhibitors (1 mM PMSF, 1 *μ*M/mL aprotinin, 1 *μ*g/mL leupeptin, and 1 mM Na_3_VO_4_). Equal amounts of proteins were separated by sodium dodecyl sulfate-polyacrylamide gel electrophoresis (SDS-PAGE) on 8–13% gels and transferred to nitrocellulose membranes (Bio-Rad). Membranes were blocked with 5% skim milk in Tris-buffered saline, followed by incubation with primary antibodies for 3 h at room temperature (RT). Blots were developed with peroxidase-conjugated secondary antibodies, and immunoreactive proteins were visualized with enhanced chemiluminescence (ECL) reagents, according to the manufacturer's recommendations (Amersham Biosciences, Buckinghamshire, UK). Experiments were repeated at least three times.

### 2.5. RNA Isolation, Reverse Transcription, Semiquantitative Reverse Transcriptase-Polymerase Chain Reaction (RT-PCR), and Quantitative Real-Time PCR

Total RNA was isolated from A549 cells using an RNeasy Kit (Qiagen, Valencia, CA, USA). cDNA was synthesized from total RNA by reverse transcription (RT) for semiquantitative RT-PCR and real-time PCR. Semiquantitative PCR was carried out using a GeneAmp PCR System 9700 (Applied Biosystems, Foster City, CA, USA), and real-time PCR was performed using SYBR Green (Fermentas, Burlington, ON, Canada) and a Chromo4 Four-Color Real-Time PCR Detector (Bio-Rad) according to the manufacturer's guidelines. Thermocycling conditions for semiquantitative RT-PCR and real-time PCR were as follows: one cycle of denaturation at 95°C for 3 min, followed by 30 cycles of amplification at 95°C for 15 s and 56°C for 20 s. The following primer pairs were used:* Nrf2*, 5′-GAGAGCCCAGTCTTCATTGC-3′ (forward) and 5′-TGCTCAATGTCCTGTTGCAT-3′ (reverse);* HO-1*, 5′-AAGATTGCCCAGAAAGCCCTGGAC-3′ (forward) and 5′-AACTGTCGCCACCAGAAAGCTGAG-3′ (reverse);* NQO-1*, 5′-GGGCAAGTCCATCCCAACTG-3′ (forward) and 5′-GCAAGTCAGGGAAGCCTGGA-3′ (reverse);* Srx1*, 5′-CAACTGCAGCGAGAGACCAT-3′ (forward) and 5′-AAAGAGAATGCACCCCTGCT-3′ (reverse); and* GAPDH*, 5′-ACCACAGTCCATGCCATCAC-3′ (forward) and 5′-TCCACCACCCTGTTGCTGTA-3′ (reverse). Experiments were repeated at least three times.

### 2.6. Measurement of ROS Generation

Intracellular ROS levels were measured using 2′,7′-dichlorodihydrofluorescein diacetate (H_2_DCFH-DA; Invitrogen Life Technologies, Gaithersburg, MD, USA), as previously described [17]. Briefly, cells with or without TGF-*β* and hypoxia/reoxygenation treatment were treated with 10 *μ*M H_2_DCFH-DA for 20 min and then washed with PBS before trypsinization. After detaching with trypsin, cells were collected, washed, and resuspended in PBS. ROS inhibition was evaluated by treating cells with 2 mM N-acetyl-l-cysteine (NAC) 1 h before the treatment. Fluorescence was detected using a FACSort flow cytometer (Becton Dickinson, BD PharMingen, San Diego, CA, USA) at excitation/emission wavelengths of 488/525 nm or a fluorometer (Victor 2; Perkin Elmer) at 480/530 nm.

### 2.7. RNA Interference

Human Nrf2-small interfering RNA (siRNA), HO-1-siRNA, and nontargeting scramble-siRNA were purchased from Santa Cruz Biotechnology. Cells were transfected with these siRNAs using Lipofectamine RNAi Max (Invitrogen) according to the manufacturer's recommendations. The final concentration of the siRNAs was 10 nM.

### 2.8. Cell Viability Assay

Cell proliferation was assessed by 3-(4,5-dimethylthiazol-2-yl)-2,5 diphenyl tetrazolium bromide (MTT) assay (Sigma-Aldrich) according to the manufacturer's recommendations. A549 cells were plated in 96-well plates at a density of 1 × 10^4^ cells/well in triplicate, treated with 1 ng/mL TGF-*β*, and incubated with hypoxic medium for 2 h before IR with 4 Gy (^137^Cs *γ*-radiation; 2.55 Gy/min) followed by reoxygenation. To determine cell viability, MTT (0.5 mg/mL) was added to each well for 3 h, and the absorbance was measured at 540 nm using a microplate reader (Thermo Labsystems, Multiskan EX, Waltham, MA).

### 2.9. Statistical Analysis

Results are shown as means ± standard deviations (SDs). Data were analyzed with two-tailed Student's *t*-tests. Differences with *P* values of less than 0.05 were considered statistically significant.

## 3. Results

### 3.1. Nrf2 and Its Target Genes Were Induced following Treatment with TGF-*β* and Hypoxia/Reoxygenation

**Figure 1:**
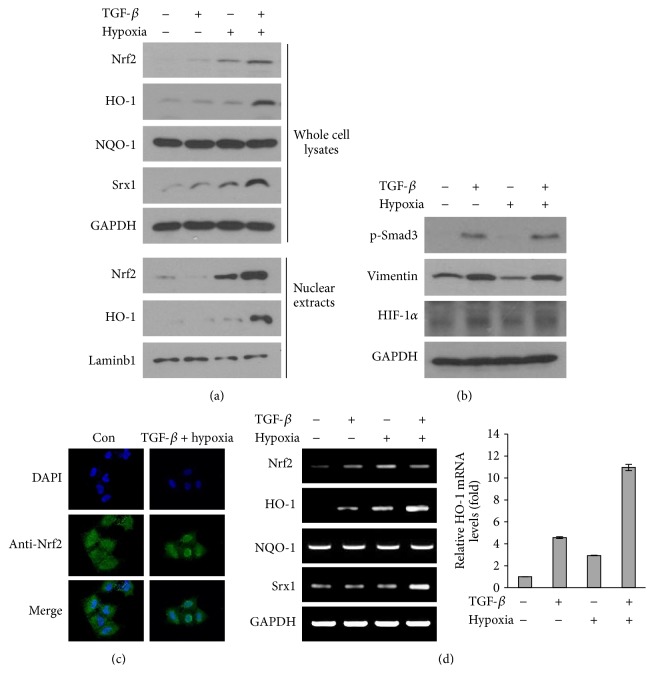
Combined treatment with TGF-*β* and hypoxia induced the expression of Nrf2 and its downstream targets in a human non-small cell lung cancer cell line. A549 cells were treated with 1 ng/mL TGF-*β* and incubated under hypoxia for 2 h, followed by reoxygenation. Cells were prepared 8 h after cotreatment with TGF-*β* and hypoxia/reoxygenation. ((a) and (b)) Protein levels of Nrf2, HO-1, NQO-1, and Srx1 (a) and levels of phospho-Smad3, vimentin, and HIF-1*α* (b) were determined by western blotting. GAPDH was used as a loading control. (c) Proteins were detected with antibodies for Nrf2 and visualized by confocal microscopy. (d) RT-PCR analysis was performed to analyze the expression of* Nrf2* mRNA and its target genes. The expression of* HO-1* mRNA was determined by real-time PCR.

Because TGF-*β* and unstable oxygenation of the tumor microenvironment are critical factors influencing tumor malignancy, we examined the effects of combined TGF-*β* treatment and hypoxia and reoxygenation for fluctuating oxidation in A549 cells on oxidative stress responses, including Nrf2. The combined treatment significantly induced Nrf2 protein expression in nuclear extracts and whole cell lysates and upregulated the expression of target proteins downstream of Nrf2 (i.e., HO-1 and Srx-1) at 8 h (Figures 1(a) and S1A). A549 cells were treated with TGF-*β* and incubated for different time points under hypoxia and reoxygenation. Nrf2 expression was increased to a greater extent after treatment with TGF*β* plus hypoxia with reoxygenation compared with that after TGF*β* plus hypoxia without reoxygenation (Figure S1B in Supplementary Material available online at http://dx.doi.org/10.1155/2016/6823471). No changes in NQO-1 expression were observed. In addition, immunofluorescence confocal microscopy showed that Nrf2 was localized to both the cytoplasm and nucleus in control cells but was prominently translocated to the nucleus following combined treatment with TGF-*β* and hypoxia/reoxygenation (Figure 1(c)). We next determined whether TGF-*β* and hypoxia/reoxygenation affected the transcript levels of* Nrf2* and its downstream targets. We found that the expression of* HO-1* and* Srx* mRNAs was induced 8 h after combined treatment, whereas expression of* Nrf2* mRNA was only slightly affected (Figure 1(d)). Moreover, combined treatment with TGF-*β* and hypoxia/reoxygenation did not more induce phosphorylation of Smad3 or vimentin as compared with those after TGF-*β* alone, and no changes in the expression of HIF-1*α* protein were observed (Figure 1(b)). These results suggested that TGF-*β* and unstable oxygenation promoted the stability of Nrf2 protein and enhanced its transcription activity.

### 3.2. Combined Treatment with TGF-*β* and Hypoxia/Reoxygenation Induced Positive Crosstalk between Nrf2 and EGFR

**Figure 2:**
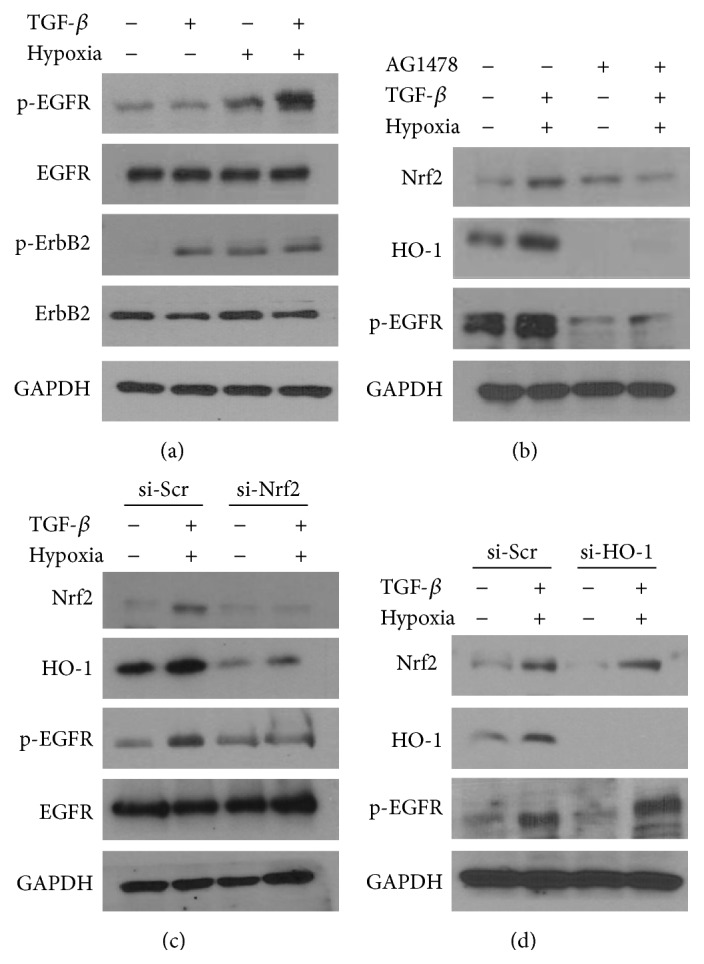
Ligand-independent phosphorylation of EGFR was induced and positively regulated by Nrf2 following combined treatment with TGF-*β* and hypoxia. (a) Cells were treated with 1 ng/mL TGF-*β* and hypoxia for 2 h, followed by reoxygenation and further incubation for 22 h. Cells were then lysed, and phospho-EGFR, total EGFR, phospho-ErbB2, and total ErbB2 levels were determined by western blotting. GAPDH was used as a loading control. (b) Cells were pretreated with 10 *μ*M AG1478 for 1 h, followed by combined treatment with 1 ng/mL TGF-*β* and hypoxia/reoxygenation. Cells were lysed 22 h later, and phospho-EGFR, Nrf2, and HO-1 levels were determined by western blotting. ((c) and (d)) Cells were transiently transfected with 10 nM of scrambled-siRNA (si-Scr), Nrf2-siRNA (c), or HO-1-siRNA (d) for 24 h. Cells were then treated with 1 ng/mL TGF-*β* and incubated with hypoxic medium for 2 h, followed by reoxygenation.

EGFR is a major oncogenic target in NSCLC. Therefore, we next examined the effects of combined treatment with TGF-*β* and hypoxia/reoxygenation on activation of EGFR. A549 cells exhibited maximum phosphorylation of EGFR at 24 h after combined treatment (Figures 2(a) and S1C), while phosphorylation of ErbB2 was increased by either TGF-*β* or hypoxia/reoxygenation alone without a significant additive increase after combined treatment. To further examine the effects of the combination treatment on activation of Nrf2 and EGFR, human NSCLC H1299 cells with wild-type Keap1 were treated with TGF-*β* and hypoxia/reoxygenation. Consistent with the results of A549 cells, the combined treatment increased Nrf2 protein expression and EGFR phosphorylation in H1299 cells (Figure S1D). We hypothesized that combined treatment with TGF-*β* and hypoxia/reoxygenation may potentiate Nrf2 activation through induction of EGFR activation; thus, we assessed the effects of blockade of EGFR signaling on Nrf2 activation. Expression of Nrf2 was inhibited by treatment with AG1478, an EGFR-tyrosine kinase inhibitor (TKI), in these cells (Figure 2(b)). We next determined the effects of inhibition of Nrf2 on EGFR phosphorylation. Interestingly, ligand-independent phosphorylation of EGFR was significantly inhibited by transfection with Nrf2-siRNA following combined treatment with TGF-*β* and hypoxia/reoxygenation in A549 cells (Figure 2(c)). We then determined whether knockdown of HO-1 regulated EGFR phosphorylation or Nrf2 activation under the same experimental conditions in A549 cells. However, neither EGFR nor Nrf2 were inhibited by transfection with HO-1-siRNA in these cells (Figure 2(d)). These results indicated that the combined treatment induced positive crosstalk between Nrf2 and EGFR.

### 3.3. Nrf2 Was Activated by Akt Signaling under Tumor Microenvironment-Like Conditions

**Figure 3:**
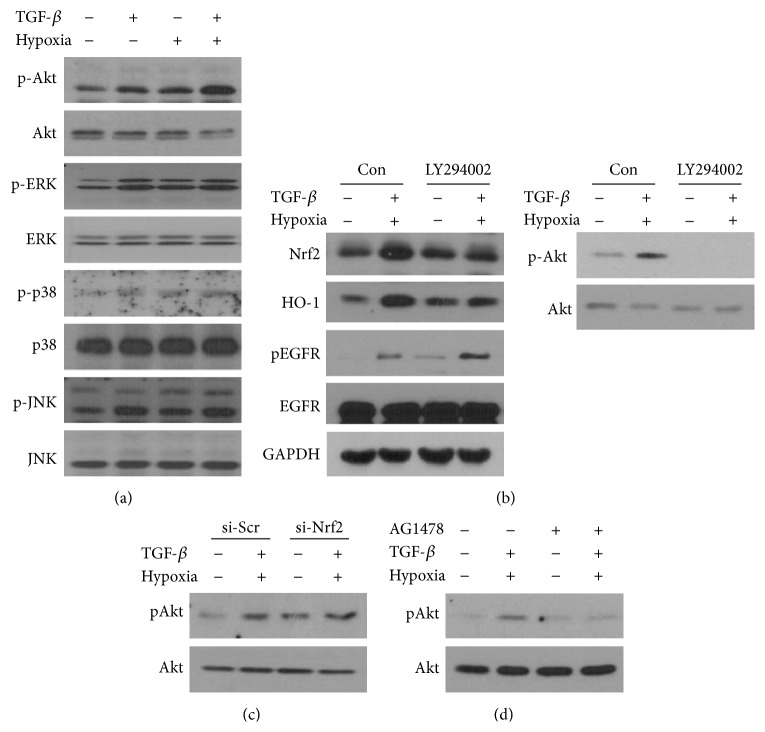
Phosphorylation of Akt was involved in Nrf2 activation in the TGF-*β* and hypoxic microenvironment in A549 cells. (a) Cells were treated with 1 ng/mL TGF-*β* and hypoxia for 2 h, followed by reoxygenation for 1 h. Phospho-Akt, total Akt, phospho-ERK, total ERK, phospho-p38 MAPK, total p38 MAPK, phospho-JNK, and total JNK levels were determined by western blotting. (b) Cells were pretreated with 10 *μ*M LY924002 for 1 h and then subjected to combined treatment with 1 ng/mL TGF-*β* and hypoxia/reoxygenation. Cells were lysed 24 h later, and Nrf2, HO-1, and phospho-EGFR levels were determined by western blotting. Phosphorylated Akt and total Akt were detected at 3 h under the same experimental conditions. (c) Cells were transiently transfected with 10 nM of scramble-siRNA (si-Scr) or Nrf2-siRNA (si-Nrf2) for 24 h, followed by combined TGF-*β* and hypoxia/reoxygenation. Phospho-Akt and total Akt levels were determined by western blotting. (d) Cells were pretreated with 10 *μ*M AG1478 for 1 h, followed by combined treatment with TGF-*β* and hypoxia/reoxygenation.

Because activation of both EGFR and Nrf2 is involved in mitogen-activated protein kinase (MAPK) and phosphoinositol 3-kinase- (PI3K-) Akt signaling [18, 19], we examined changes in the phosphorylation of Akt, ERK, p38 kinase, and JNK after treatment with TGF-*β* and hypoxia/reoxygenation in A549 cells. Phosphorylation of Akt was increased, while that of MAPKs was unaffected by the combined treatment (Figure 3(a)). To clarify whether Akt activation contributed to the activation of Nrf2 or EGFR, A549 cells were treated with LY294002, a PI3K inhibitor, before combined treatment with TGF-*β* and hypoxia/reoxygenation. LY294002 inhibited TGF-*β* and hypoxia/reoxygenation-induced Nrf2 protein expression but slightly induced EGFR phosphorylation. Since Akt was highly phosphorylated at 3 h and returned to an unphosphorylated state at 24 h after treatment with TGF-*β* plus hypoxia/reoxygenation, we confirmed that LY294002 inhibited Akt phosphorylation at 3 h under the same experimental conditions (Figure 3(b)). Moreover, while Nrf2 knockdown by siRNA did not inhibit Akt phosphorylation, treatment with AG1478 did block Akt phosphorylation in this cell model (Figures 3(c) and 3(d)).

### 3.4. Induction of Nrf2 and EGFR Signaling in the Tumor Microenvironment Was Associated with ROS Accumulation

**Figure 4:**
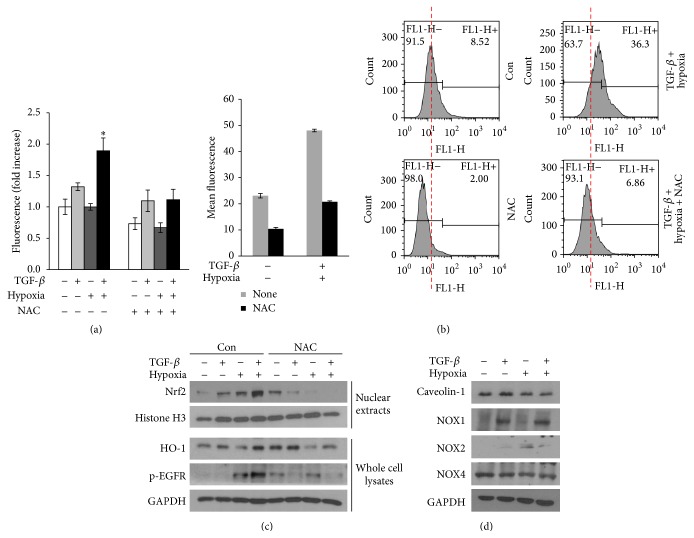
Generation of ROS by TGF-*β* and hypoxia regulated Nrf2 and EGFR activation. ((a) and (b)) A549 cells were treated with TGF-*β* and hypoxia for 2 h followed by reoxygenation for 1 (a) or 22 h (b). After reoxygenation, ROS levels were measured using a fluorometer (a) or FACS analysis (b). Cells were pretreated with 1 mM N-acetyl-l-cysteine (NAC) for 1 h before TGF-*β* and hypoxia/reoxygenation. Data are representative of at least three independent experiments. ^*∗*^ *P* < 0.05 versus the corresponding value for control ROS. (c) Nuclear extracts or whole cells were lysed after cotreatment with TGF-*β* and hypoxia for 2 h, followed by reoxygenation for 6 h. Nrf2, HO-1, and phospho-EGFR levels were then determined by western blotting. Histone H3 and GAPDH were used as loading controls. (d) Cells were treated as described in (c), and whole cell lysates were analyzed by western blotting for caveolin-1, NOX1, NOX2, and NOX4.

Previous studies have shown that single treatment with TGF-*β* or hypoxia/reoxygenation promotes ROS production [20, 21]. Therefore, we measured intracellular ROS levels at 1 and 22 h after reoxygenation following a 2 h treatment with TGF-*β* and hypoxia using the fluorescent indicator H_2_DCFH-DA. Combined treatment of A549 cells with TGF-*β* and hypoxia/reoxygenation significantly increased ROS levels at 3 h after treatment (i.e., 2 h of treatment with TGF-*β* plus hypoxia followed by a 1 h reoxygenation period; Figure 4(a)) for analysis with H_2_DCFH-DA and at 24 h after treatment (i.e., 2 h of treatment with TGF-*β* plus hypoxia followed by a 22 h reoxygenation; Figure 4(b)) for analysis by FACS; quantification of these changes showed that the combined treatment caused 1.8- and 2.1-fold increases in ROS levels compared with the control, respectively. The increase in ROS induced by TGF-*β* and hypoxia/reoxygenation was blocked by treatment with NAC, a general free radical scavenger, which restored ROS levels to the value of the control cells. To determine the role of ROS in the activation of Nrf2/EGFR signaling, A549 cells were treated with or without NAC prior to TGF-*β* treatment and hypoxia/reoxygenation, and Nrf2, phosphorylated EGFR, and HO-1 expression levels were analyzed by western blotting (Figure 4(c)). Treatment of cells with NAC suppressed the observed increase in Nrf2 and HO-1 protein levels and EGFR phosphorylation following combined treatment with TGF-*β* and hypoxia/reoxygenation. These results suggested that the combined treatment contributed to ROS generation, which played a role in mediating Nrf2/EGFR signaling in A549 cells. Previous studies have shown that NOX family proteins enhance ROS production in the presence of TGF-*β* or under hypoxic conditions [21–23]. Additionally, decreased expression of caveolin-1 (Cav-1) is involved in NOX-dependent ROS generation [24]. Therefore, we next determined changes in the expression levels of Cav-1, NOX1, NOX2, and NOX4 proteins after the combined treatment. Cav-1 was slightly downregulated under hypoxia/reoxygenation alone and after TGF-*β* plus hypoxia/reoxygenation; however, this difference was not statistically significant. Similarly, no significant changes in the expression of NOX1, NOX2, or NOX4 proteins were observed (Figure 4(d)).

### 3.5. Nrf2 Contributed to the Radioresistance of NSCLC Cells under Tumor Microenvironment-Like Conditions

**Figure 5:**
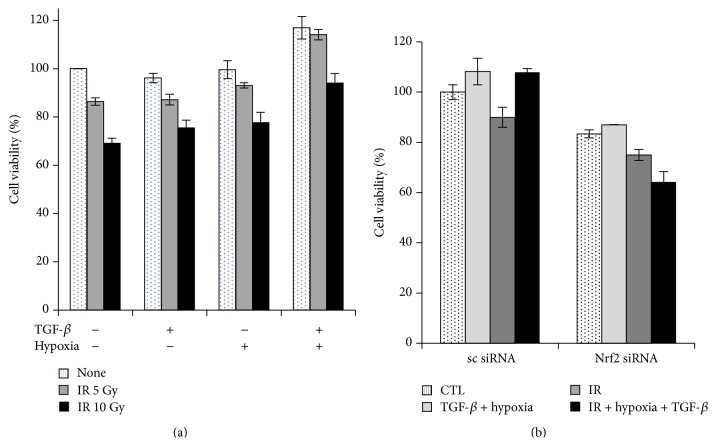
Combined treatment with TGF-*β* and hypoxia enhanced resistance to radiation in A549 cells. (a) Cells were pretreated with 1 ng/mL TGF-*β* and incubated with hypoxic medium for 2 h before IR (5 or 10 Gy), followed by reoxygenation. MTT assays were performed 48 h after IR. (b) Cells were transiently transfected with 10 nM of scramble-siRNA or Nrf2-siRNA for 24 h and were then subjected to combined treatment with TGF-*β* and hypoxia/reoxygenation. MTT assays were performed 48 h after IR.

To assess the effects of tumor microenvironment-like conditions using combined TGF-*β* and unstable oxygenation on cancer cell proliferation, A549 cells were treated with TGF-*β* under hypoxic conditions for 2 h and then exposed to IR 5 Gy or 10 Gy under normoxic conditions. MTT assays showed that TGF-*β* plus hypoxia/reoxygenation slightly enhanced cell proliferation and abolished IR-induced cell death as compared with observations in untreated control cells (Figure 5(a)). Because Nrf2 is known to confer cancer cells with a radioresistant phenotype, we next investigated the role of Nrf2 in IR-mediated cell death following combined treatment with TGF-*β* and hypoxia/reoxygenation. Following knockdown of Nrf2 by siRNA in A549 cells, cells appeared to be sensitized cells to IR under combined TGF-*β* treatment and hypoxia/reoxygenation (Figure 5(b)).

## 4. Discussion

Although Nrf2 plays a beneficial role in normal cells, recent studies have suggested that Nrf2 functions as an oncogene in tumor progression. As a solid tumor rapidly grows, it becomes hypoxic due to abnormal vasculature, which is unable to supply sufficient nutrients and oxygen to the tumor tissues. Aberrant tumor microvessels may result in dynamic changes in oxygenation due to closing and reopening of vessels, thereby leading to hypoxia and reoxygenation [25]. In addition to fluctuations in oxygen concentrations, the tumor microenvironment is also characterized by the presence of TGF-*β*, which plays a prominent role in tumor progression by inducing changes in the epithelial phenotype and stromal environment [26–28]. Based on this background, we aimed to elucidate the roles of redox molecules in tumor malignancy and the tumor microenvironment, mimicked* in vitro* by combined treatment with TGF-*β* and hypoxia/reoxygenation. Interestingly, HIF-1*α* was not altered under these experimental conditions. This may be explained by the observation that reoxygenation after hypoxia may abolish hypoxia-induced HIF-1*α* expression; thus, the shorter incubation time after reoxygenation may affect HIF-1*α* expression. In addition, Smad signaling was also not augmented under the tumor microenvironment-like conditions. We observed crosstalk between Nrf2 and EGFR signaling in this model, providing important insights into cancer progression.

Nrf2 expression is maintained at low basal levels by constant degradation by the ubiquitin- (Ub-) proteasome pathway (UPP) under quiescent conditions; however, in cancer cells harboring dysfunctional Keap1, such as A549 cells, Nrf2 is constitutively activated. A recent study demonstrated that Nrf2 is activated by stimulation with EGF, an EGFR ligand, in cells carrying both wild-type* EGFR* and* Keap1* genes. In contrast, Nrf2 is not regulated by EGFR signaling in Keap1-mutant cells, suggesting that Keap1 is essential for Nrf2/EGFR activation in ligand-dependent EGFR signaling [18]. Moreover, EGFR directly binds to and phosphorylates Keap1 to reduce the ability of Keap1 to sequester Nrf2, leading to Nrf2 activation [29]. However, in our study, we found that Nrf2 and EGFR were activated in A549 cells expressing wild-type EGFR and lacking a functional* Keap1* gene (i.e., having the mutation G333C in the* Kelch* domain), suggesting that Nrf2 could be activated in a Keap1-independent manner. Moreover, siRNA-mediated knockdown of Nrf2 or inhibition of EGFR by AG1478 inhibited EGFR phosphorylation and Nrf2 protein expression, respectively, indicating that there was positive crosstalk between Nrf2 and EGFR under the tumor microenvironment-like conditions created by treatment with TGF-*β* and hypoxia/reoxygenation without direct EGF ligand stimulation or activation of the EGFR-Keap1 axis. To the best of our knowledge, this is the first study demonstrating that inhibition of Nrf2 inhibited EGFR phosphorylation and that AG1478 inhibited Nrf2 expression and signaling. Interestingly, our results showed that Nrf2 itself, rather than Nrf2 downstream signaling, affected EGFR phosphorylation.

PI3K/Akt activation contributes to cancer cell proliferation and metastasis, driving cancer progression [30]. We found that Akt signaling regulated TGF-*β* and hypoxia/reoxygenation-induced Nrf2 activation, but not EGFR signaling. Moreover, EGFR regulated Akt, while Nrf2 did not. According to previous studies, TGF-*β* or intermittent hypoxia can induce ROS production in many types of cancer cells. Indeed, TGF-*β*-induced ROS activates MAPK and Smad signaling, which promote the EMT in renal disease. Moreover, TGF-*β* induces NOX4-dependent ROS production, which contributes to the EMT and cells migration in breast cancer cells. Malec et al. demonstrated that intermittent hypoxia (exposure to 2 h of hypoxia 2-3 times, interrupted by 2 h intervals) promotes HIF-1*α* expression through the Nrf2-Trx1 axis in response to NOX1-mediated ROS generation [21–23]. Consistent with these data, we found that the combined treatment with TGF-*β* and hypoxia/reoxygenation induced ROS generation. Additionally, NAC treatment abolished the activation of Nrf2 and EGFR under these conditions. However, we did not detect increased expression of NOX family proteins in the combined treatment as compared to TGF-*β* or hypoxia/reoxygenation alone. Therefore, NOX1 and NOX4 may partially contribute to ROS generation and subsequent Nrf2/EGFR activation. Notably, in this experiment, reoxygenation after TGF-*β* plus hypoxia produces free radicals; thus, this mechanism may contribute further to the observed results. Additional studies are needed to elucidate the mechanisms affecting ROS generation in this context. Based on these observations, we suggest that TGF-*β* plus hypoxia/reoxygenation-induced ROS is required for the activation of Nrf2 signaling and EGFR. Recently, Tao et al. demonstrated that K-RAS (both ectopic wild-type K-RAS and a K-RAS mutant harboring G12D) and activation of ERK can increase* Nrf2* gene transcription through the TPA response element located in the regulatory region of the* Nrf2* gene, subsequently increasing its transcriptional activity and promoting chemoresistance [31]. Although A549 cells harbor mutant K-RAS, our data demonstrated that phosphorylation of ERK was not induced in A549 cells with the combined treatment as compared with TGF-*β* or hypoxia/reoxygenation alone (Figure 2(a)). To exclude the possibility that the activated K-RAS-ERK pathway increased activation of Nrf2 and EGFR, we examined the effects of ERK inhibition on activation of Nrf2 and EGFR. Treatment with PD98059, a MEK inhibitor, slightly inhibited protein expression of Nrf2 and HO-1 at the basal level but did not affect the protein expression of Nrf2 and HO-1 or the phosphorylation of EGFR after combined hypoxia and TGF-*β* treatment (data not shown). Although K-RAS and ERK are closely related to Nrf2 and EGFR signaling, we believe that Akt plays a key role in the hypoxia- and TGF-*β*-mediated Nrf2-EGFR pathway in A549 cells.

In this study, we demonstrated that TGF-*β* and hypoxia/reoxygenation enhanced A549 cell proliferation compared with the effects of TGF-*β* or hypoxia/reoxygenation alone. Moreover, these cells showed resistance to IR under the tumor microenvironment-like conditions. Interestingly, siRNA-mediated knockdown of Nrf2 significantly inhibited the proliferation of A549 cells, even in the presence of TGF-*β* and hypoxia/reoxygenation. These findings suggested that Nrf2 acted a key player regulating cancer cell proliferation and resistance to IR.

**Figure 6:**
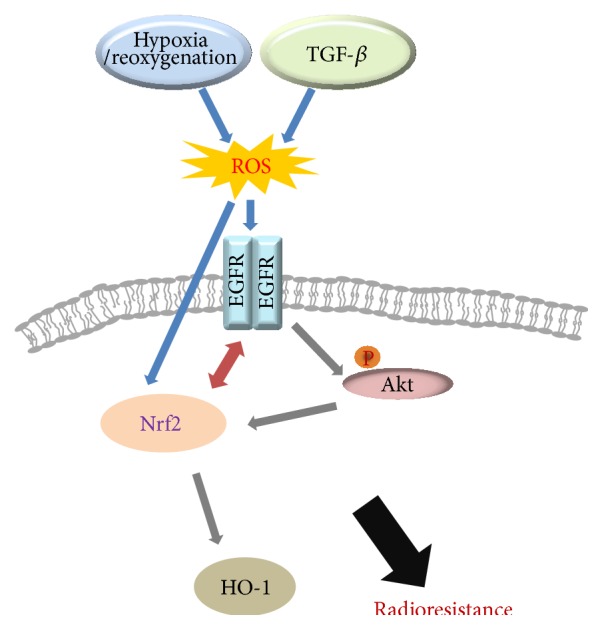
Schematic diagram showing the proposed mechanism for regulation of Nrf2-EGFR-Akt signaling in the TGF-*β* and hypoxic microenvironment.

In conclusion, our findings demonstrated that crosstalk between Nrf2 and EGFR played a pivotal role in cancer progression following combined treatment with TGF-*β* and hypoxia/reoxygenation, as shown in the schematic in Figure 6. To the best of our knowledge, this is the first study demonstrating that Keap1-independent Nrf2 activation and ligand-independent EGFR activation were involved in positive crosstalk in A549 lung cancer cells. Moreover, our data supported that Nrf2 activation may be directly related to EGFR phosphorylation/activation not through a mechanism involving the EGFR-/Keap1 interaction during tumor cancer progression. Additionally, Keap1-independent activation of Nrf2 has been shown to be associated with resistance to EGFR-TKI despite expression of the wild-type* EGFR* gene in NSCLC cells [18]. Thus, Nrf2 may be a promising molecular target for enhancement of chemo- and radiotherapeutic effects in the context of oxidative stress and a metastatic microenvironment.

## Supplementary Material

Figure S1. Combined treatment with TGF-β and hypoxia induced the Nrf2 expression and EGFR phosphorylation in A549 cells. (A) A549 cells were treated with 1 ng/mL TGF-β and incubated under hypoxia for 2 h, followed by reoxygenation for 1 or 6 h. Cells were prepared 8 h after cotreatment with TGF-β and hypoxia/reoxygenation for western blot analysis. (B). A549 cells were treated with 1 ng/mL TGF-β and incubated under hypoxia for the indicated time periods (1, 2, 4, 7, or 8 h), followed by reoxygenation (1, 4, 6, or 7 h), and cell lysates were analyzed by western blot. (C) Cells were treated for the indicated times as described in Figure S1A to detect phospho-EGFR. (D) H1299 cells were treated with 1 ng/mL TGF-β and incubated under hypoxia for 2 h, followed by reoxygenation for 6 h. Cells were prepared 8 h after the treatment for western blot analysis.
