# Supplementary material for: TGF-β and Hypoxia/Reoxygenation Promote Radioresistance of A549 Lung Cancer Cells through Activation of Nrf2 and EGFR
Source: Oxid Med Cell Longev. 2016 Jan 20;2016:6823471. doi: 10.1155/2016/6823471 (PMC4745286; doi:10.1155/2016/6823471)
Supplement: Supplementary file 1 — Figure S1. Combined treatment with TGF-β and hypoxia induced the Nrf2 expression and EGFR phosphorylation in A549 cells. (A) A549 cells were treated with 1 ng/mL TGF-β and incubated under hypoxia for 2 h, followed by reoxygenation for 1 or 6 h. Cells were prepared 8 h after cotreatment with TGF-β and hypoxia/reoxygenation for western blot analysis. (B). A549 cells were treated with 1 ng/mL TGF-β and incubated under hypoxia for the indicated time periods (1, 2, 4, 7, or 8 h), followed by reoxygenation (1, 4, 6, or 7 h), and cell lysates were analyzed by western blot. (C) Cells were treated for the indicated times as described in Figure S1A to detect phospho-EGFR. (D) H1299 cells were treated with 1 ng/mL TGF-β and incubated under hypoxia for 2 h, followed by reoxygenation for 6 h. Cells were prepared 8 h after the treatment for western blot analysis. [file 6823471.f1.pdf]

Supplemental figure legend

Fig. S1. Combined treatment with TGF-β and hypoxia induced the Nrf2 expression and EGFR phosphorylation in A549 cells. (A) A549 cells were treated with 1 ng/mL TGF-β and incubated under hypoxia for 2 h, followed by reoxygenation for 1 or 6 h. Cells were prepared 8 h after cotreatment with TGF-β and hypoxia/reoxygenation for western blot analysis. (B). A549 cells were treated with 1 ng/mL TGF-β and incubated under hypoxia for the indicated time periods (1, 2, 4, 7, or 8 h), followed by reoxygenation (1, 4, 6, or 7 h), and cell lysates were analyzed by western blot. (C) Cells were treated for the indicated times as described in Fig. S1A to detect phospho-EGFR. (D) H1299 cells were treated with 1 ng/mL TGF-β and incubated under hypoxia for 2 h, followed by reoxygenation for 6 h. Cells were prepared 8 h after the treatment for western blot analysis.

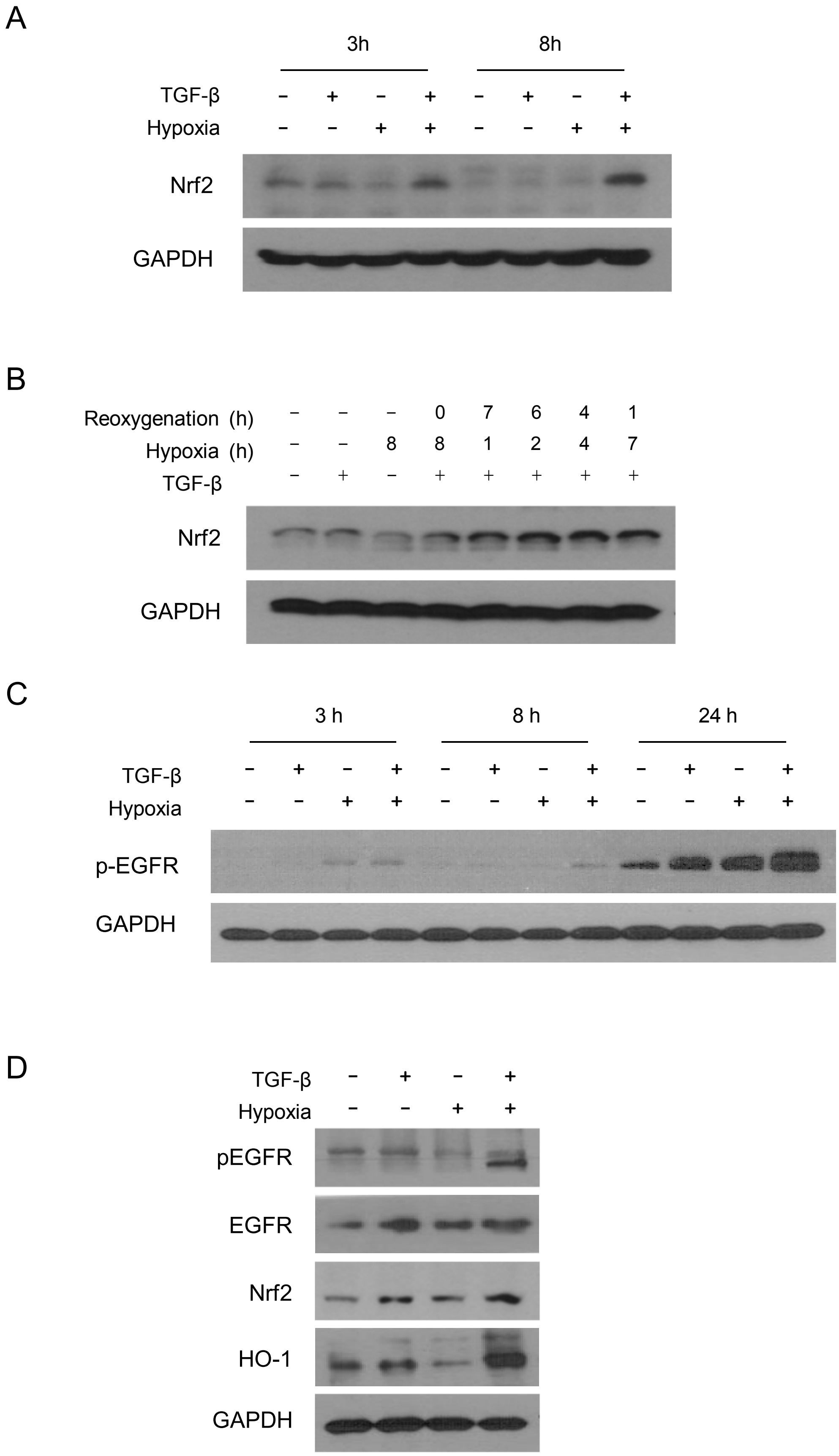

Supplemental Figure 1
